# Supplementary material for: Striped nanoscale phase separation at the metal–insulator transition of heteroepitaxial nickelates
Source: Nat Commun. 2016 Nov 2;7:13141. doi: 10.1038/ncomms13141 (PMC5097133; doi:10.1038/ncomms13141)
Supplement: Supplementary Information — Supplementary Figures 1-5 and Supplementary Note 1 [file ncomms13141-s1.pdf]

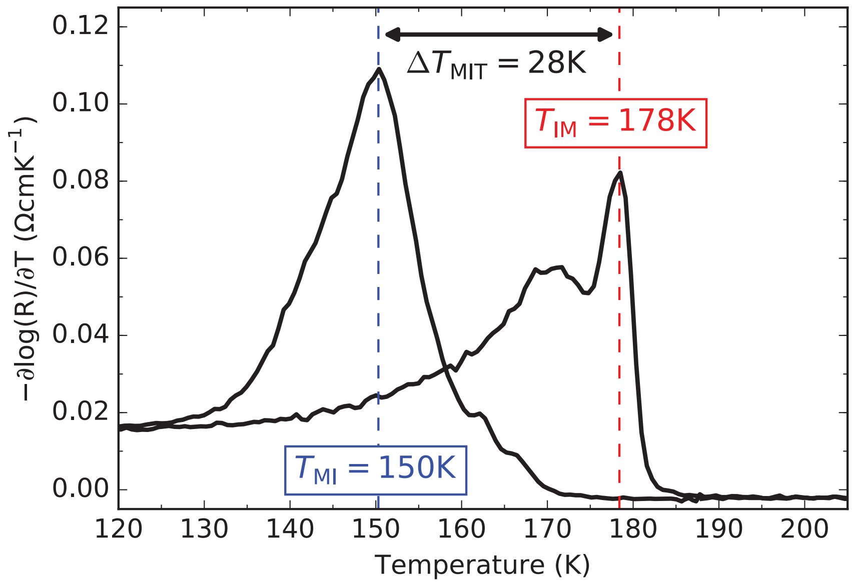

Supplementary Figure 1: **Logaritmic derivative of the resistivity from the transport measurement.** The transition temperatures $T_{\mathrm{MI}} = 150\,\mathrm{K}$ and $T_{\mathrm{IM}} = 178\,\mathrm{K}$ are defined as the peaks of $-\partial \log R/\partial T$ during a cooling and warming cycle, respectively. From the peaks separation the hysteresis width $\Delta T_{\mathrm{MIT}} = 28\,\mathrm{K}$ is extracted.

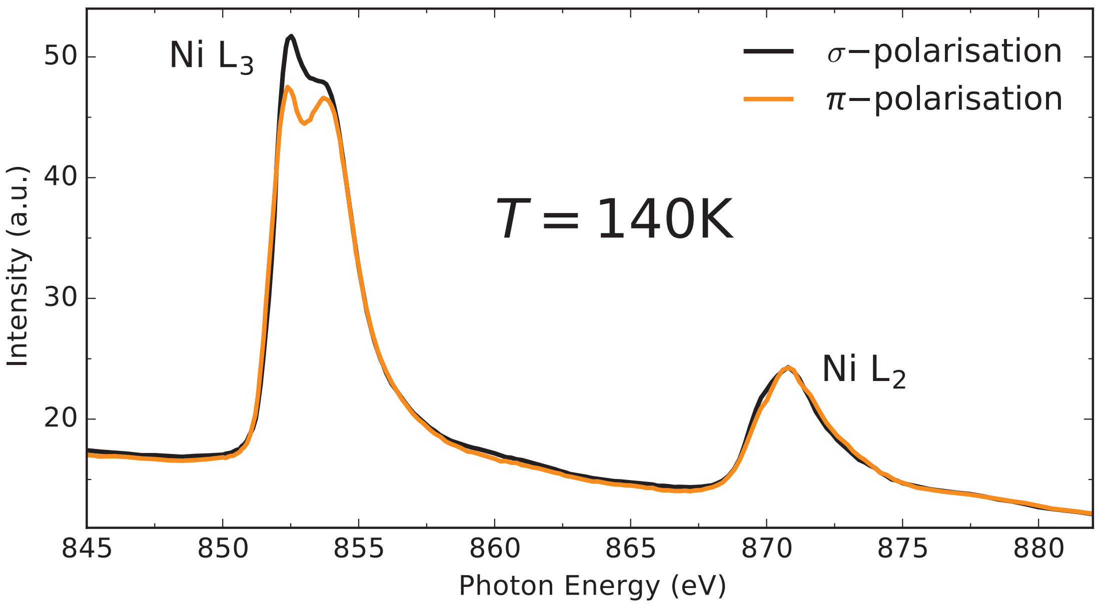

Supplementary Figure 2: **Full-range Nickel XAS taken with different X-ray linear polarisations.** A small dichroism in the XAS is observed, stemming from a combined effect of orbital symmetry and charge ordering, consistent with previous reports [Tung et al., Phys. Rev. B 88, 205112 (2013)]. When considering the spatial distribution of dichroic signal measured by PEEM, however, we do not observe any spatial variation compared to our noise level, for any photon energy and sample temperature. As described in the main text for the case of σ-polarisation, it is also possible to acquire PEEM measurements at 852.0 eV and 852.7 eV with π-polarised X-rays. Calculating their difference pixel-by-pixel, it is possible to construct equivalent PEEM images to the one presented in the main text. We overall used σ-polarisation as it provides the most intense absorption peak, thus determining a better signal-to-noise ratio.

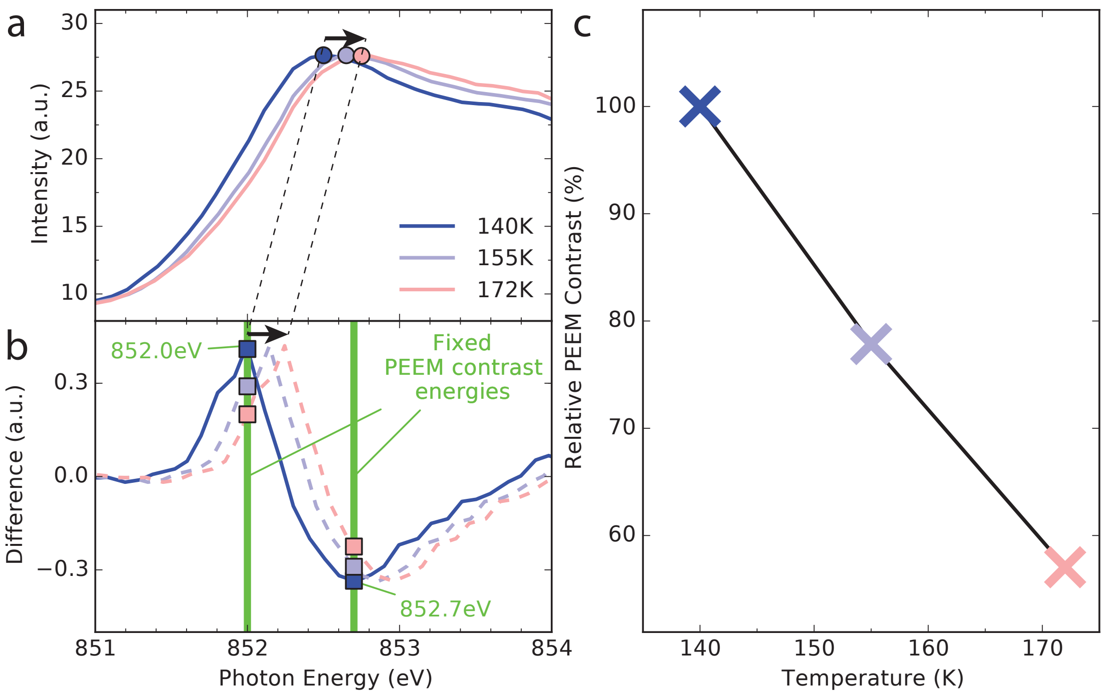

Supplementary Figure 3: **Temperature dependence of PEEM imaging contrast.** (a) close-up of the shift in photon energy of Ni L_3 XAS peak as a function of temperature, measured over the full field of view, from fig. 2a. Panel (b) indicates how the XAS difference of fig. 2c changes in temperature and (c) shows the relative change in PEEM contrast.

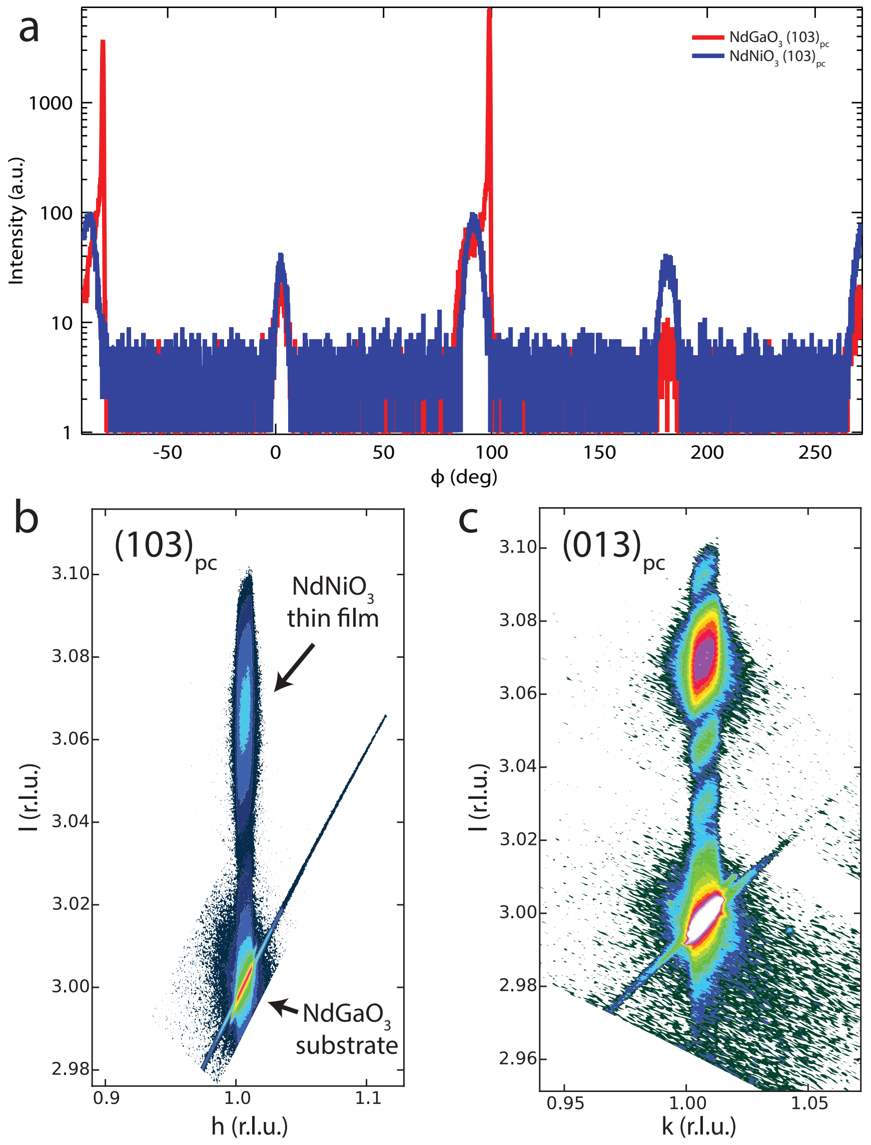

Supplementary Figure 4: **Additional X-ray diffraction measurements of the $NdNiO_3$ film.** (a) ϕ-scan recorded along the $(103)_{pc}$ direction. The aligned scans of the $NdGaO_3$ substrate and $NdNiO_3$ thin film confirm a crystallographically oriented heteroepitaxy. In (b) and (c) reciprocal space maps of the $NdNiO_3$ film around the $(103)_{pc}$ and $(013)_{pc}$ reflections. The in-plane intensity distribution of the $NdNiO_3$ film and $NdGaO_3$ substrate are confined to the same value of reciprocal lattice vector $h = 1$ in (b) and $k = 1$ in (c). The film is thus coherently oriented to the substrate lattice. From the position of the peaks we extract $c_{\mathrm{NdGaO_3}} = 3.86\,\mathrm{nm}$ and $c_{\mathrm{NdNiO_3}} = 3.77\,\mathrm{nm}$, consistent with previous reports [Catalan G. et al., Phys. Rev. B 62, 7892 (2000)].

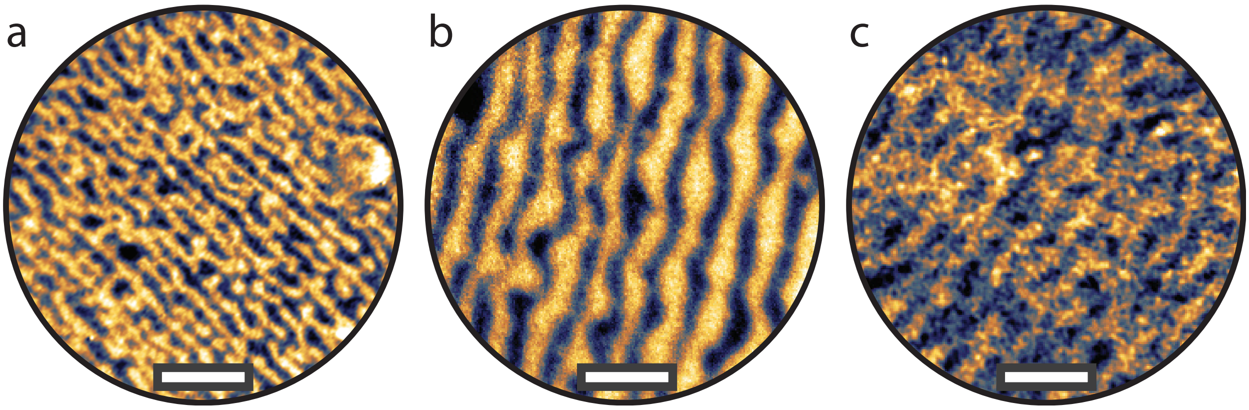

Supplementary Figure 5: **PEEM images taken on 3 different samples:** (a) sample discussed in the main text, (b) sample with larger surface terraces, and (c) another sample. The insulating domains orientation and size changes from sample to sample according to direction and size of the surface terraces. This remarks the determining contribution of heteroepitaxy in driving the formation of the insulating phase at the metal-insulator transition.

Supplementary Note 1

In the main text we used two fixed photon energies (852.0 eV and 852.7 eV) to construct PEEM images at all temperatures. Because the Ni L_3 XAS peaks change in temperature, the specific pair of energies that maximises the PEEM contrast is slightly different for each different temperature. This would have involved repeating the analysis presented in fig. 2c for all the temperature points, which is a very long measurement. Therefore we decided to slightly sacrifice the contrast at higher temperatures in order to perform faster acquisitions, thus improving the number of data points taken during the temperature ramps. In fig. 3 we estimate the amount of contrast-loss as a function of temperature, demonstrating it has a negligible impact on our analysis. In particular, in fig. 3a we present a close-up of the shift in photon energy of Ni L_3 XAS peak as a function of temperature, measured over the full field of view, from fig. 2a. The most intense peak of the multiplet (solid circles) shifts about 0.2 eV. At a given temperature, the domain resolved XAS spectra of the metallic and insulating areas are slightly displaced as shown in fig. 2c. The difference between these spectra at 140 K is maximum at 852.0 eV and 852.7 eV, therefore the PEEM contrast at 140 K is optimal at such energies. Figure 3b indicates how the XAS difference of fig. 2c changes in temperature under the assumption that both the metallic and insulating spectra evolve similarly. The dashed lines in fig. 3b are rigid shifts of the 140 K curve (solid line) by amounts extracted from the peak positions in fig. 3a. In fig. 3c we estimate the relative change in PEEM contrast by calculating the vertical difference between the squares in fig. 3b normalised with respect to 140 K. We see that, in the studied temperature range (140 K to 170 K), the PEEM contrast loss is smaller than 50%. This allowed to track the evolution of the insulating phase at all temperatures, supporting the validity of our assumptions.
